# The Global Campaign turns 18: a brief review of its activities and achievements

**DOI:** 10.1186/s10194-022-01420-0

**Published:** 2022-04-21

**Authors:** Timothy J. Steiner, Gretchen L. Birbeck, Rigmor H. Jensen, Paolo Martelletti, Lars Jacob Stovner, Derya Uluduz, Matilde Leonardi, Jes Olesen, Zaza Katsarava

**Affiliations:** 1 Department of Neuromedicine and Movement Science, Norwegian University of Science and Technology, Edvard Griegs gate, Trondheim, Norway; 2 Division of Brain Sciences, Imperial College London, London, UK; 3 UTH Neurology Research Office, University of Zambia, Lusaka, Zambia; 4 Department of Neurology, University of Rochester, Rochester, NY USA; 5 Department of Neurology, Danish Headache Centre, University of Copenhagen, Rigshospitalet-Glostrup, Glostrup, Denmark; 6 Department of Clinical and Molecular Medicine, Sapienza University, Rome, Italy; 7 Department of Neurology and Clinical Neurophysiology, Norwegian Advisory Unit On Headaches,, St Olavs Hospital, Trondheim, Norway; 8 Neurology Department, Istanbul University Cerrahpaşa School of Medicine, Istanbul, Turkey; 9 Public Health and Disability Unit, Fondazione IRCCS Istituto Neurologico C Besta, NeurologyMilan, Italy; 10 Centre of Neurology, Geriatric Medicine and Early Rehabilitation, Evangelical Hospital, Unna, Germany; 11 Medical Faculty, University of Essen, Essen, Germany

**Keywords:** Headache, Burden, Health care, Structured headache services, Public health, Change management, Project management, Implementation, Global Campaign against headache

## Abstract

The Global Campaign against Headache, as a collaborative activity with the World Health Organization (WHO), was formally launched in Copenhagen in March 2004. In the month it turns 18, we review its activities and achievements, from initial determination of its strategic objectives, through partnerships and project management, knowledge acquisition and awareness generation, to evidence-based proposals for change justified by cost-effectiveness analysis.

## Background

In 1996, two of us (TJS and JO) commenced a dialogue with the World Health Organization (WHO) in Geneva. Our messages were straightforward: that headache disorders were ubiquitous, prevalent and disabling – and to a very large extent treatable. These were WHO’s criteria for priority.

There was global persistence of substantial and largely unmitigated headache-attributed burden, with universal barriers to care maintaining wide gaps between need for treatment and its provision. The roots of these health-care failures were established in educational failures. From poor understanding, headache was accorded little priority despite clear evidence that this was wrong. It was wrong from a public-health perspective, and it was wrong economically in view of the very high financial costs of headache disorders. Educational failures blocked awareness, so that this evidence was unseen, or ignored.

According to its mandate to promote health through universal health coverage [1], we argued, WHO should take action to reduce this burden.

Thus, more than 25 years ago, were sown the seeds of the Global Campaign against Headache. They did not instantly spring to life. WHO requested empirical evidence to support these arguments, which took time to muster but was in due course presented in WHO’s own Global Burden of Disease study (GBD) 2000. The *World Health Report 2001*, portraying the estimates of GBD2000 and assimilating all evidence that we could then gather on migraine-attributed burden, ranked migraine among the top 20 causes of disability worldwide [2]. The Global Campaign was the outcome, formally launched in Copenhagen in March 2004 [3, 4].

The way forward was not clear: the evident scale of the problem mandated an effective response, but also stood in the way of any solution. Needs analysis revealed then, as it does now, the potentially daunting demands for headache-related health care of the very large numbers who might benefit from it [5–14]. The approach required was broad, its distant target ambitious. It called for strategic partners, of whom WHO would be the most important [3, 4, 15–26], along with the Institute for Health Metrics and Evaluation (IHME) at the University of Washington (see later). The path took time to construct [3, 4, 16].

*Lifting The Burden* (LTB), a non-governmental organisation registered in UK, was created in 2009 to formalise the strategic partnerships, particularly with WHO. In 2011, LTB was invited into Official Relations with WHO [18], a recognition of its track record of achievement already [16], and of the importance among WHO’s priorities accorded to the Campaign [22–25].

## The Global Campaign

### Strategy and tactics

The Global Campaign is an agent for change, not merely an advocate of change. Strategically, it was conceptualised in three stages, each of these aligned with one of three strategic objectives, themselves directionally determined by change-management theory (Table 1).

**Table 1:** The three strategic objectives of the Global Campaign

Strategic objective		Purpose	Action
1	Knowledge for awareness	Establish what it is that requires change	Adduce and collate evidence of the scope and scale of the global burden of headache
2	Awareness for action	Agitate to create desire for change	Promote awareness, among politicians, health-care providers, employers, schools and the general public, of headache disorders as remediable causes of public ill health and disability, and high financial cost
3	Action for change	Propose and justify the change to be instigated	Develop evidence-based, adaptable recommendations for intervention, justified by cost-effectiveness analysis

Tactically, in line with standard project-management methodology, the Campaign was implemented through reduction, breaking it into small component activities to be reassembled, ultimately, into a coherent whole [4]. At this level, the Campaign depended on academic collaborations established as a network throughout the world, and on tactical partnerships, most importantly with the Norwegian University of Science and Technology (NTNU), its academic base since 2009 [27], with the International Headache Society, European Headache Federation and European Brain Council, and with the *Journal of Headache and Pain*, its official journal.

### Knowledge for awareness

Stage 1 of the Campaign recognised that the scope and scale of a problem must be clearly known before its remedy could be envisaged.

When, early on, all existing data on the burden of headache were collated, Western Europe and North America were far better represented than elsewhere, and migraine far better than other headache disorders [6]. What was then known of headache covered less than half the world’s population, among whom only half of the burden attributable to headache was estimated with any reliability.

Filling these large knowledge gaps was the first priority (Table 1), requiring a series of new population-based burden-of-headache studies. Most of these would be in low- and middle-income countries, and promised to be methodologically and financially challenging. Therefore, LTB brought together an international expert consensus group to establish standardised methodology and questionnaire [28–32]. Adult studies using these have now been conducted in all world regions: African (Ethiopia [33, 34] and Zambia [35, 36], Benin, Cameroon and Mali [not yet published], and Malawi in a HIV-positive population [37]); American (Brazil [38] and Peru [not yet published]); Eastern Mediterranean (Pakistan [39–41], Saudi Arabia [42, 43] and Morocco [not yet published]); European (Georgia [44–47], Lithuania [48], Russia [49–52] and, within the Eurolight project, eight countries of western Europe [53–59]); South East Asia (India south [Karnataka State] [60–64], Nepal [65–74] and India north [Delhi and National Capital Territory Region] [not yet published]); Western Pacific (China [75–81] and Mongolia [82, 83]). Schools-based child and adolescent studies began later, again with development and testing of new methodology [84, 85]. Studies have completed data collection in Austria [86], Ethiopia [87], Lithuania [88, 89] and Turkey [90], and in Benin, Iran, Mongolia, Serbia and Zambia [not yet published]. Others have commenced or are planned in Brazil, Cambodia, Cameroon, Estonia, Georgia, Nepal and Spain, but are interrupted by the SARS-CoV-2 pandemic.

These studies inform local policy as well as global knowledge. To the extent that they have been conducted in low- and middle-income countries, they have enhanced research capacity in these countries [21] as a collateral benefit. Among other such benefits are a broader understanding of the full spectrum of headache-attributed burden, which goes far beyond symptom burden and disability [10–12, 14, 22, 29, 30, 32, 51, 54, 58, 84, 85, 90–94].

Two databases under construction at NTNU are capturing the individual-participant data (*ie*, primary data) from all LTB population-based studies, with sub-datasets describing sampling and other methodology as attributes of the main datasets. Ultimately, following development and imposition of quality controls, these will be available as free goods for academic purposes, as are all Global Campaign products.

### Awareness for action

In its second stage, conducted almost in parallel with the first, the Campaign has used the knowledge it gathered to raise awareness – among people with headache, health-care providers and health-policy makers in particular. Making the case for change through evidence-based argument was the second priority (Table 1), with the key message that headache was not a health problem only of industrialised high-income countries – an historical misperception.

The Global Campaign could not have achieved this objective without its strategic partners.

On the one hand, the *Atlas of headache disorders and resources in the world, 2011* was published jointly by WHO and LTB, collating data from more than 100 countries [22]. On the other, LTB has collaborated with IHME [95] since 2005, providing expert advice, health-state descriptions (on which disability weights are based [96]) and epidemiological data to inform all iterations of GBD from GBD2010 onwards [97–106]. GBD incorporates the findings of all population-based studies contributing to global knowledge, including LTB’s (all of these recently reviewed [107]). Migraine and tension-type headache (TTH) were included in GBD2010. Medication-overuse headache (MOH) entered GBD2013 (as 18^th^ highest cause of disability), but, from GBD2016 onwards, has been included as a *sequela* of migraine (73%) or TTH (27%), and its consequences attributed accordingly [103]. Increasingly better informed, GBD has advanced the ranking of headache disorders generally and of migraine in particular among the global causes of disability: the latter from 19^th^ in GBD2000 [2] to second (first among young women) in GBD2019 [108–115].

There has been no better means of fostering awareness of headache as a public-health priority. The *Atlas*, along with its political messages of “worldwide neglect of a major cause of public ill-health and … the inadequacies of responses to it in countries throughout the world” [22], was distributed directly by WHO to the world’s Ministries of Health. The highly respected GBD data are a public good, directly shared with WHO and all governments, and available to health-policy makers everywhere [95].

### Action for change

**Fig. 1:**
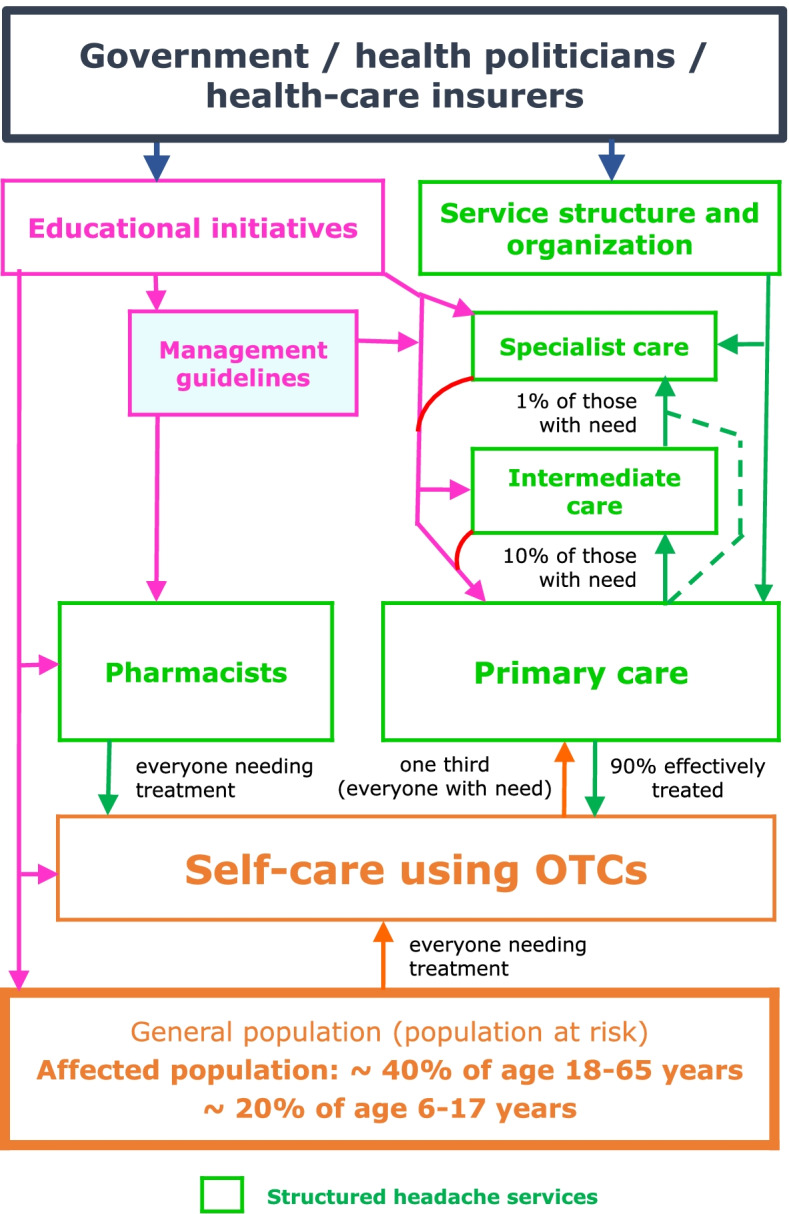
Adaptable template for structured headache services based in primary care and supported by educational initiatives and management aids, with expected patient flows (from [128])

In its third stage, the Campaign has proposed the health-care solution to headache, a template for structured headache services adaptable to local needs and resources (Fig. 1), and supported it with evidence-based scientific, political and economic arguments [22, 116–128].

Many issues came up for consideration. How and where should headache services be organised? How and by whom, and with what level of resource allocation, should they be delivered? And what were the reasons for whatever were the answers to these? What features of a headache service contributed to quality? These might include technical success (would it function well at a practical level?), uptake (would it be used?), clinical outcomes (would it make patients better?), user satisfaction (would patients, and health-care providers, be happy with it?), cost-effectiveness (would it be affordable within the health-care or wider societal economies?) and equity (would there be equal access for all with equal need?). Which of these were most important, and from whose perspective? How were they measured?

The first and foremost requirement of a putative solution is to dismantle the barriers to care [129–133]. Structured headache services achieve this to a large extent through their base in accessible primary care (Fig. 1), where patients generally prefer to receive care [128]. They do not deny the role of specialist care, but this cannot be the focus or principal provider of ubiquitous, efficient, cost-effective and equitable care [134]. Placing the burden of care largely into the hands of non-specialists calls for some additional education [135–137], and a range of clinical management supports [138, 139]: diagnostic aids based on the *International Classification of Headache Disorders* [139], management principles [139, 140], and outcome measures to aid initial assessment and follow-up [141–146]. Public education is required to dismantle other barriers: banishing stigma, and promoting self-efficacy, in which people understand their headache disorders and seek and utilise care appropriately [139, 147–151].

All of these materials must be translated if they are to succeed transnationally and transculturally, following protocols to ensure conceptual equivalence [139, 152–154]. The outcome measures created by LTB [144, 145] are already freely available in 13 languages [155].

Quality evaluation of headache services first required an understanding of how “quality” should be defined in this context [156, 157]. Subsequently, field-tested indicators of quality were needed, with methods and instruments to measure quality as defined [158–162]. This remains work in progress, with bench-marking the next step.

### Paying for change

Finally, in a world of competing demands and scarce resources, health-policy makers require evidence of the economic value of intervention if they are to be persuaded to invest accordingly. Empirical evidence of this for headache was limited outside the very restricted context of clinical trials, in which gains from use of specific drugs had been small and unconvincing. Efficiency is one key to economic value: poor knowledge and understanding of headache lead to misdiagnosis, mismanagement and poor outcomes, wasting health-care resources while often making the initial problem worse. Through avoidance of this wastage, by education, presently allocated resources can achieve much more than they do [22].

But the major economic opportunity is through reduction, by better care delivered more widely, in the very heavy consequential (lost-productivity) costs of headache [163–166]. Economic evaluation of structured headache services has used LTB’s empirical burden-of-headache data from population-based studies around the world [33–83], but first required development of a new metric (hours lived with disability [HLDs]), applicable to all forms of treatment, care and care-delivery systems as opposed to comparisons of single-modality treatments [167, 168]. By this measure, structured headache services as proposed [128], properly implemented with educational supports in place, are not merely cost-effective in a range of economies, in terms of health gained per dollar spent [169], but cost saving at societal level in many [170, 171].

## Conclusion

The Global Campaign against Headache is a coherent programme of multiple constituent parts, its path determined by its strategic objectives, its activities guided by its values [4] and its progress towards its objectives dependent on a global network of partners. After 18 years, the once-distant target is now visible on the near horizon, but there is a lot more to be done [172, 173]. The Campaign’s aspirational vision, from outset, has been of “a future world in which headache disorders are recognised everywhere as real, disabling, and deserving of medical care. In this world, all who need headache care have access to it, without artificial barriers” [174]. The Global Campaign can inform, motivate and offer the means, but only governments can realise this vision.

## Data Availability

Not applicable.

## Abbreviations

GBD: Global Burden of Disease (study)
HLDs: Hours lived with disability
IHME: Institute for Health Metrics and Evaluation
LTB: *Lifting The Burden*
MOH: Medication-overuse headache
NTNU: Norwegian University of Science and Technology
TTH: Tension-type headache
WHO: World Health Organization
